# Use of Essential Oils to Increase the Safety and the Quality of Marinated Pork Loin

**DOI:** 10.3390/foods9080987

**Published:** 2020-07-24

**Authors:** Lorenzo Siroli, Giulia Baldi, Francesca Soglia, Danka Bukvicki, Francesca Patrignani, Massimiliano Petracci, Rosalba Lanciotti

**Affiliations:** 1 Department of Agricultural and Food Sciences, University of Bologna, Piazza Goidanich 60, 47521 Cesena, Italy; lorenzo.siroli2@unibo.it (L.S.); giulia.baldi4@unibo.it (G.B.); francesca.soglia2@unibo.it (F.S.); francesca.patrignani@unibo.it (F.P.); m.petracci@unibo.it (M.P.); 2 Institute of Botany and Botanical Garden “Jevremovac”, Faculty of Biology, University of Belgrade, Takovska 43, 11000 Belgrade, Serbia; dankabukvicki@gmail.com; 3 Interdepartmental Center for Industrial Agri-food Research, University of Bologna, Via Quinto Bucci 336, 47521 Cesena (FC), Italy

**Keywords:** essential oil, marinating solution, pork loin, quality, safety

## Abstract

This study aimed at evaluating the effects of the addition of an oil/beer/lemon marinade solution with or without the inclusion of oregano, rosemary and juniper essential oils on the quality, the technological properties as well as the shelf-life and safety of vacuum-packed pork loin meat. The results obtained suggested that, aside from the addition of essential oils, the marination process allowed to reduce meat pH, thus improving its water holding capacity. Instrumental and sensorial tests showed that the marination also enhanced the tenderness of meat samples, with those marinated with essential oils being the most positively perceived by the panelists. In addition, microbiological data indicated that the marinated samples showed a lower microbial load of the main spoiling microorganisms compared to the control samples, from the 6th to the 13th day of storage, regardless of the addition of essential oils. Marination also allowed to inhibit the pathogens *Salmonella enteritidis*, *Listeria* *monocytogenes* and *Staphylococcus* *aureus,* thus increasing the microbiological safety of the product. Overall outcomes suggest that the oil/beer/lemon marinade solution added with essential oils might represent a promising strategy to improve both qualitative and sensory characteristics as well as the safety of meat products.

## 1. Introduction

In the past decade, global consumer demand for marinated meat products has significantly increased [1,2]. The reasons behind this scenario are mainly related to the nutritional characteristics, the extended shelf-life as well as the improvement of sensorial and textural traits of this kind of commodity [2,3]. In addition, marination technology allows to diversify meat products and, conferring them peculiar sensorial traits, to offer a broader choice to the consumers [4]. Marination is a widely used process in the meat industry consisting in the injection or immersion of meat cuts into aqueous solutions containing a wide range of ingredients such as water, salt, vinegar, lemon juice, wine, soy sauce, brine, essential oils, tenderizers, herbs, spices and organic acids [5,6]. Depending on the selected ingredients, a huge variety of marinade solutions, either alkaline or acid, exists. The firsts contain phosphates, while the seconds are usually prepared with the addition of organic acids or their salts [7,8]. Another type of marinade solution are the water/oil emulsions. Overall, the addition of marinade solutions to a meat cut is usually performed to improve the production yields (i.e., by increasing the moisture content of the product), improve the organoleptic characteristics of the final product and, eventually, limit (or at least retard) the occurrence of oxidative reactions [9,10,11]. In addition, recent studies have reported that marinade solutions including “natural” ingredients (e.g., spices, herbs, essential oils, etc.) can exert an antimicrobial effect against pathogenic and spoilage microorganisms in poultry, beef and pork meat [5,12,13]. Aside from their ability to improve the safety and the shelf-life of marinated meat [14], the utilization of ingredients such as essential oils may also enhance consumers’ willingness to buy, in light of the recent increasing attitude towards the consumption of clean-label products [15].

The use of essential oils or of their components (extracted from flowers, fruits, roots, buds and leaves through distillation processes) is widespread in the food industry, precisely in light their organoleptic, antimicrobial and antioxidant properties [16,17,18]. Within this context, a remarkable antimicrobial effect of several essential oils (included during processing) has been recently highlighted. To cite some examples, the use of rosemary essential oil (0.05%) on beef and chicken meat was found to be able to inhibit the growth of *Listeria monocytogenes*, *Escherichia coli* and *Staphylococcus aureus* [19,20]. On the other hand, the inclusion of thyme essential oil (0.08%) allowed to inhibit the growth of both spoiling microorganisms such as *Pseudomonas* spp. and pathogens such as *Staphylococcus aureus* [16]. Oregano essential oil has been found to exert antimicrobial effects against various pathogenic microorganisms such as *Escherichia coli*, *Listeria monocytogenes* and *Salmonella enteritidis* on both beef and pork meat [16]. However, it is noteworthy to mention that, as essential oils have low sensory thresholds [17], their sensory compatibility as well as their impact on the sensory profile of the final product should be carefully considered [21,22].

In addition, the flavor innovation represents a marketing strategy aimed at keeping up with the continually changing food trends [23]. Within this context, creating appealing alternatives for the consumers represents an important challenge for the meat industry. As a matter of fact, the possibility to set-up a marinade solution with typical ingredients of the Mediterranean area could certainly offer an added value to the final product and differentiate it from the alternatives currently existing on the market. In this framework, the purpose of this research was to evaluate the effect of the addition of a marinade solution composed by olive oil, beer and lemon (i.e., typical ingredients from Mediterranean area) with or without the inclusion of a mixture of essential oils on the shelf-life, the safety as well as the sensory and quality traits of vacuum-packed pork loin slices.

## 2. Materials and Methods

### 2.1. Preliminary Tests: Selection of the Marinade Solution’s Composition and Essential Oils Mixture

Preliminary tests were performed on pork loin slices (weighing about 60 g) in order to set the best combination and concentration of ingredients in the marinade solution in terms of either organoleptic traits (taste, smell, tenderness) and technological properties (absorption of the marinade solution, tenderness, color, etc.). In detail, 8 ingredients (i.e., water, lemon juice, olive oil, balsamic vinegar, red wine, white wine, beer and mustard) have been tested through different combinations and ratios as well as percentage of marinade solution (*w/w*) added to the meat slices, as reported in Table 1.

With the aim to obtain homogenous solutions, the ingredients of each combination were mixed with an Ultraturrax (IKA–WERKE, Labortechnik, Staufen, Germany) (13,000 rpm, 30 s, in ice). To each slice of pork loin, 1% NaCl (*w/w*) was added in the marinated product. The samples were placed in heat-resistant plastic bags, in which the marinating solution was directly added. The slices were then vacuum packaged (99.9%) and placed in a small-scale tumbler (model MHG-20, VakonaQualitat, Lienen, Germany) under vacuum conditions (−0.95 bar) and at a temperature of 2–4 °C. Tumbling was performed in 60 min at a speed of 20 rpm including two working cycles (25 min per cycle) and a 10 min pause cycle.

Subsequently, in order to select the combination allowing to obtain the best organoleptic properties of the product without altering its flavor, the addition of essential oils to the marinade solutions was tested. The essential oils considered during the preliminary tests were thyme, rosemary, oregano, and juniper, in different combinations and concentrations (0.02, 0.04, 0.06 and 0.08% on the final product). The evaluation was done by an untrained panel of 20 panellist taking into consideration the sensory parameters such as color, odour, overall accettability before and after cooking.

On the basis of preliminary results (data not shown), the marinade solution selected for the main experiment was composed by olive oil/beer/lemon juice (1:2:1, 10% *w/w*) with a mixture of oregano (0.02%), rosemary (0.03%) and juniper (0.03%) essential oils.

### 2.2. Ingredients and Microorganisms Used

The pork loin slices used in this work were obtained from a local retailer the same day of the trial and kept at refrigerated temperatures (4 ± 1 °C) until the analyses. The marinade solution was composed as follows: the bock style beer Moretti la rossa (7.2% ABV) (Heineken Italia S.p.A., Pollein, AO, Italy), extra virgin olive oil (Monini, Spoleto, PG, Italy) and concentrated lemon juice (LIMMI, Perugia, PG, Italy). The essential oils used in this experimentation were oregano, rosemary, and juniper (Flora, Pisa, PI, Italy).

The strains used in the challenge test trial, *Listeria monocytogenes* Scott A, *Salmonella enteritidis* E5 and *Staphylococcus aureus* SR41 belonged to the Department of Agricultural and Food Sciences of Bologna University. The strains were maintained at −80 °C before experiments and before inoculation they were cultured twice in Brain Heart Infusion broth (BHI, Oxoid Ltd. Basingstoke, UK) at 37 °C for 24 h.

### 2.3. Preparation of the Samples and Shelf-Life Trials

The experiment was carried out on a total of 81 slices of pork loin (having an average weight of 60 g), divided into 3 groups (27 slices/group) as follows:

1. Control group (non-marinated) added with 1% NaCl (C);
2. Marinade solution beer/olive oil/concentrated lemon juice (2/1/1; 10% *w/w*) (M);
3. Marinade solution beer/olive oil/concentrated lemon juice (2/1/1; 10% *w/w*) added with a mixture of essential oils (oregano 0.02%, rosemary 0.03% and juniper 0.03% essential oils) (M + E).

As previously mentioned, the marinade solution was realized by mixing bock style beer, concentrated lemon juice and extra virgin olive oil at a 2:1:1 ratio using an Ultraturrax (IKA–WERKE, Labortechnik, Staufen, Germany) (13,000 rpm, 30 s, in ice). Part of this solution was used for samples belonging to the experimental group M, while the remaining was added of a mixture of essential oils (0.08% of the final weight) consisting of oregano (0.02%), rosemary (0.03%) and juniper (0.03%) and included in the samples M + E. Each pork loin slice (about 60 g), was added of 1% NaCl, calculated on the final weight of the marinated product. Subsequently, the samples were placed in heat-resistant plastic bags, in which the marinating solution was directly added, with the only exception of the samples belonging to the control group to which only 1% NaCl was included. The amount of marinade solution added to the samples corresponded to 10% (*w/w*) of the final product. The slices were then vacuum packaged (99.9%) and placed in a small-scale tumbler (model MHG-20, VakonaQualitat, Lienen, Germany) under vacuum conditions (−0.95 bar) and at a temperature of 2–4 °C. Tumbling was performed in 60 min at a speed of 20 rpm including two working cycles (25 min per cycle) and a 10 min pause cycle. The vacuum-tumbled loin slices were then stored at 4 °C and used for analytical determinations after 3, 9 and 15 days of storage.

#### 2.3.1. pH

The pH of the samples was determined by taking an aliquot of meat (avoiding fat and connective tissue) according to Jeacocke [24]. About 2.5 g of finely chopped meat were homogenized for 30 s by Ultraturrax in 25 mL of a solution 5 mM of sodium iodoacetate and 150 mM of KCl at pH 7.0. The pH was determined by pH meter (mod. Jenway 3510; Electrode 924001, Cole-Parmer, Stone, UK) previously calibrated. The pH determination was performed after 3, 6 and 15 days of refrigerated storage on raw meat samples.

#### 2.3.2. Color

Color was assessed by a Minolta^®^ CR-400 colorimeter (Milan, Italy), previously calibrated using a standard white ceramic tile, in standardized illuminant (C) and observation angle (0° with respect to an area of 8 mm in diameter) conditions. The CIELAB system [25] was utilized and the parameter of lightness (L*), redness (a*) and yellowness (b*) were used to objectively define color. The color determination was performed, for each group, after 3, 9 and 15 days of refrigerated storage on raw meat samples.

#### 2.3.3. Marinade Uptake

Marinade uptake (i.e., the ability of meat to bind the saline solution added) was calculated by the difference in weight of the samples before and after the marination process. The amount of marinade solution absorbed was calculated as a percentage of the initial weight of the meat sample, according to the formula:

Marinade uptake (%) = [(Weight after marination − Initial weight)/Initial weight] × 100

#### 2.3.4. Cooking Loss

After 3, 9 and 15 days of storage, samples were cooked in a in a stone grill (model GL-33, Fimar, Rimini, Italy) in standardized conditions (200 °C, 190 s) and the cooking loss (amount of liquid lost after cooking) was calculated as a percentage of the initial weight of the sample according to the formula:

Cooking loss (%) = [(Raw weight − Cooked weight)/Raw weight] × 100

#### 2.3.5. Shear Force

Shear force was assessed by a texture analyzer TA-HDi 500 (Stable Micro System, Godalming, Surrey, UK) equipped with a 5-kg load cell and a Warner-Bratzler shear probe. From each cooked sample, sub-samples (having the dimension of 4 × 1 × 0.5 cm) were excised and placed inside the load cell. The resulting shear force was expressed as kg/cm^2^.

#### 2.3.6. Sensory Analysis

Panel tests were performed after 3, 9 and 15 days of refrigerated storage on cooked samples in order to test their visual appearance, olfactory acceptability and taste. The analysis was carried out by 20 untrained panelists who evaluated on a 1 to 5 scale the following parameters: meat odor intensity, spicy odor intensity, color intensity, flavor intensity, tenderness, overall assessment and finally favorite sample.

#### 2.3.7. Microbiological Analysis

During storage at 4 °C, the cell count over time of lactic acid bacteria, yeasts, total aerobic mesophilic bacteria, total aerobic psychrotrophic bacteria, *Pseudomonas* spp. and *Brochotrix thermosphacta* was evaluated by plate counting in specific agar media. Aerobic mesophilic and psychotrophic bacteria were detected on Plate Count Agar (PCA, Oxoid Ltd., Basingstoke, UK), lactic acid bacteria on de Man Rogosa and Sharpe Agar (MRS, Oxoid Ltd. Basingstoke, UK) with added 0.05% cycloheximide (Sigma-Aldrich, St. Louis, US), yeasts on Sabouraud Dextrose Agar (SAB, Oxoid Ltd. Basingstoke, UK), added to 0.02% chloramphenicol (Sigma-Aldrich, St. Louis, US), *Pseudomonas* spp. on Pseudomonas Agar Base (PAB, Oxoid Ltd. Basingstoke, UK) supplemented with Pseudomonas CFC selective agar supplement (Oxoid Ltd. Basingstoke, UK) and *Brochotrix thermosphacta* on STAA Agar base (Oxoid Ltd. Basingstoke, UK) supplemented with STAA selective supplement (Oxoid Ltd. Basingstoke, UK). To perform microbiological analyses, 10 g of meat sample were diluted into 90 mL of physiological solution (0.9% (*w/v*) NaCl), homogenized by a BagMixer 400 P (Interscience, St Nom la Bretèche, France), followed by serial dilution in physiological solution. The MRS agar plates were incubated 24 h at 37 °C, the PCA plates for the detection of psychrotrophic bacteria were incubated at 10 °C for 7 days, all the other agar media were incubated at 30 °C for 24–48 h.

### 2.4. Challenge-Test Trials

The preparation of marinated pork loin was done similarly to what reported in paragraph 2.3. The experiment was carried out on a total of 60 slices of pork loin (having an average weight of 60 g), divided into 3 groups (20 slices/group). Three groups of samples were obtained:

1. Control group (non-marinated) + pathogens (*L. monocytogenes*, *S. enteritidis* and *S. aureus*), (C+P) inoculated at a level of 4.0 log CFU/g;
2. Marinade solution beer/olive oil/concentrated lemon juice (2/1/1) used at 10% + pathogens (*L. monocytogenes*, *S. enteritidis* and *S. aureus*), (M + P) inoculated at a level of 4.0 log CFU/g;
3. Marinade solution beer/olive oil/concentrated lemon juice (2/1/1) used at 10%; added with essential oils (oregano 0.02%, rosemary 0.03% and juniper 0.03% essential oils) + pathogens (*L. monocytogenes*, *S. enteritidis* and *S. aureus*), (M + E + P) inoculated at a level of 4.0 log CFU/g.

*Listeria monocytogenes* Scott A, *Salmonella enteritidis* E5 and *Staphylococcus aureus* SR231, used in the challenge test belongs to the Department of Agricultural and Food Sciences (DISTAL, University of Bologna) collection. The bacterial strains were cultured overnight two times in Brain Heart Infusion (Oxoid Ltd., Basigstone, UK) at 37 °C. The pathogens were directly inoculated on the loin slices through 0.5 mL of physiological solution for the control group, while for the groups M + P and M + E + P were added to the marinating solution before the addition to the product. The inoculum was done in order to have an initial cell load of the pathogens, on the product, of approximately 4.0 log CFU/g. After the addition of the marinating solution the product was packaged and churned as reported in paragraph 2.3. The samples were stored at 4 °C and used for microbiological analyses immediately after the marinating and after 3, 6, 9, 13 and 15 days.

### 2.5. Microbiological Analysis

During the storage microbiological analyses were performed in order to detect the cell loads of the inoculated *L. monocytogenes*, *S. enteritidis* and *S. aureus*. Specifically, the entire slice of loin (about 60 g) was placed in sterile bags and added with sterile physiological solution in a 1:2 (*w/w*) ratio and then homogenized for 2 min by a BagMixer 400 P (Interscience, St Nom la Bretèche, France) followed by serial dilution in physiological solution. *L. monocytogenes*, *S. enteritidis* and *S. aureus* were detected in specific selective agar media. Listeria Selective Agar (LSA, Oxoid Ltd., Basingstoke, UK) supplemented with Listeria selective supplement (SR0140, Oxoid Ltd., Basingstoke, UK) for the enumeration of *L. monocytogenes*; Bismuth Sulphite Agar (BSA, Oxoid Ltd., Basingstoke, UK) for the detection of *S. enteritidis,* while Baird-Parker Agar base (BPA, Oxoid Ltd., Basingstoke, UK) added with Egg Yolk Tellurite Emulsion (SR0054, Oxoid Ltd., Basingstoke, UK) for the enumeration of *S. aureus*. The agar plates were then incubated at 37 °C for 24 h.

### 2.6. Statistical Analysis

Data were analyzed using the one-way ANOVA option of Statistica software (version 8.0; StatSoft., Tulsa, Oklahoma, USA) in order to test the effect of the addition of a marinade solution (with or without the inclusion of essential oils) at each sampling time (3, 9 and 15 days). Following, mean values were separated through Tukey honest significant difference (HSD) test, by considering a significance level of *p* < 0.05.

## 3. Results

### 3.1. Shelf-Life Trials

#### 3.1.1. pH and Color

**Figure 1:**
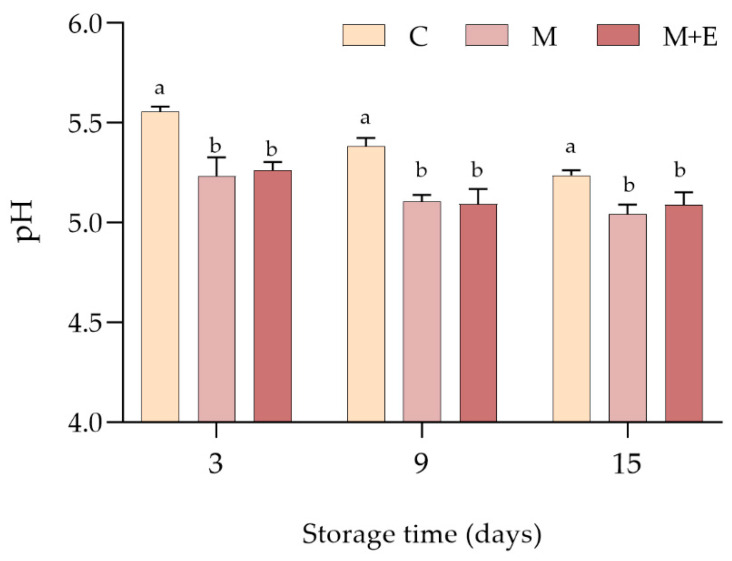
Average pH values of non-marinated (C), marinated (M) and marinated with essential oils (M + E) pork loin slices at 3, 9 and 15 days of refrigerated storage. Data represent means ± SD. a, b = average values lacking a common letter significantly differ among the same sampling time.

As reported in Figure 1, at each sampling time, control samples showed significantly higher pH than the marinated ones (*p* < 0.05) which, in their turn, exhibited similar values. A slight decrease in pH following refrigerated storage was observed for all the experimental groups, with C samples showing the greatest pH decline. In more detail, control samples exhibited an average pH decrease of 0.32 units, while M and M + E decreased of 0.19 and 0.17, respectively.

**Figure 2:**
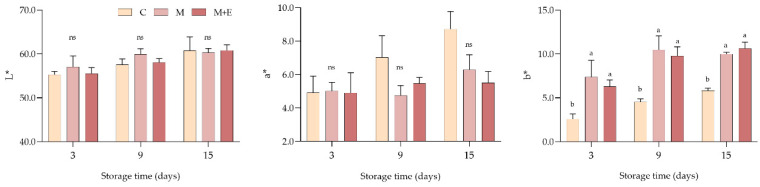
Average lightness (L*), redness (a*) and yellowness (b*) values of non-marinated (C), marinated (M) and marinated with essential oils (M + E) pork loin slices at 3, 9 and 15 days of refrigerated storage. Data represent means ± SD. a, b = average values lacking a common letter significantly differ among the same sampling time. At the same storage time, ns indicates no significant differences among the samples.

Results concerning the evolution of color parameters (lightness—L*, redness—a*, yellowness—b*) during the refrigerated storage are reported in Figure 2. Overall, regardless the storage time, no significant differences were found either in L* or a* values among the experimental groups. Although these differences were not statistically significant, non-marinated samples showed noticeably higher a* values at both 9 and 15 days of storage. On the contrary, marination treatment exploited a remarkable effect on yellowness (b*): at each storage time, both M and M + E exhibited significantly higher b* values if compared to the control (*p* < 0.05).

#### 3.1.2. Marinade Uptake and Cooking Loss

**Figure 3:**
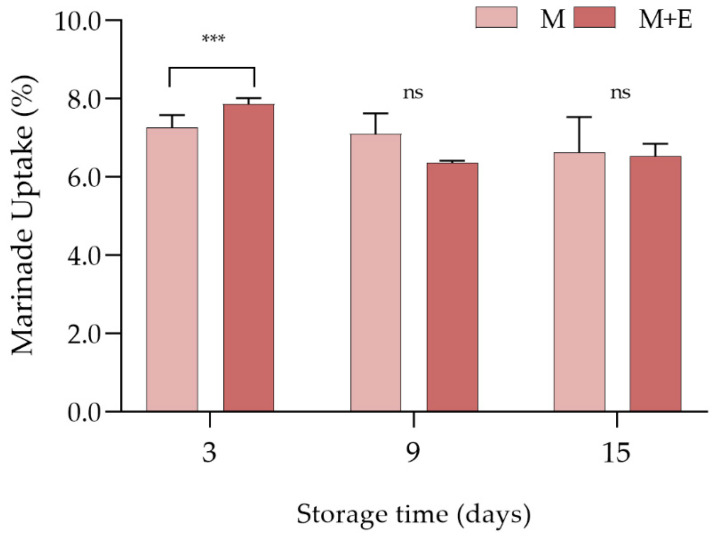
Average marinade uptake (%) values of marinated (M) and marinated with essential oils (M + E) pork loin slices at 3, 9 and 15 days of refrigerated storage. Data represent means ± SD. *** = *p* < 0.001. At the same storage time, ns indicates no significant differences among the samples.

Data concerning the marinade uptake during the refrigerated storage are shown in Figure 3. Albeit any difference has been detected among the experimental groups at 9 and 15 days of storage, at day 3, a significantly (*p* < 0.05) higher marinade uptake has been observed in M + E samples in comparison to M (7.8 vs. 7.3%, respectively).

**Figure 4:**
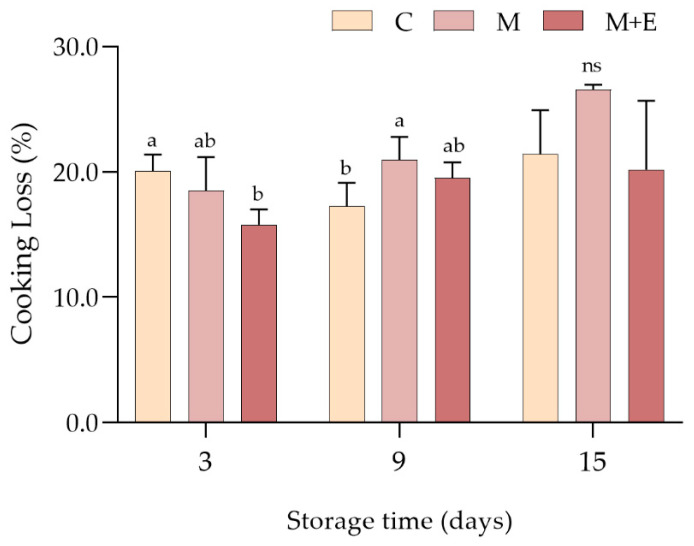
Average cooking loss values (%) of non-marinated (C), marinated (M) and marinated with essential oils (M + E) pork loin slices at 3, 9 and 15 days of refrigerated storage. Data represent means ± SD. a, b = average values lacking a common letter significantly differ among the same sampling time. At the same storage time, ns indicates no significant differences among the samples.

Results concerning the cooking losses at different storage times are shown in Figure 4. After 3 days of refrigerated storage, C (non-marinated samples) showed significantly higher liquid losses if compared to M + E samples (*p* < 0.001), while M group exhibited intermediate values. However, different results were observed at day 9 with the C group showing significantly lower values if compared to M, whereas no significant differences were found at day 15.

#### 3.1.3. Shear Force

**Figure 5:**
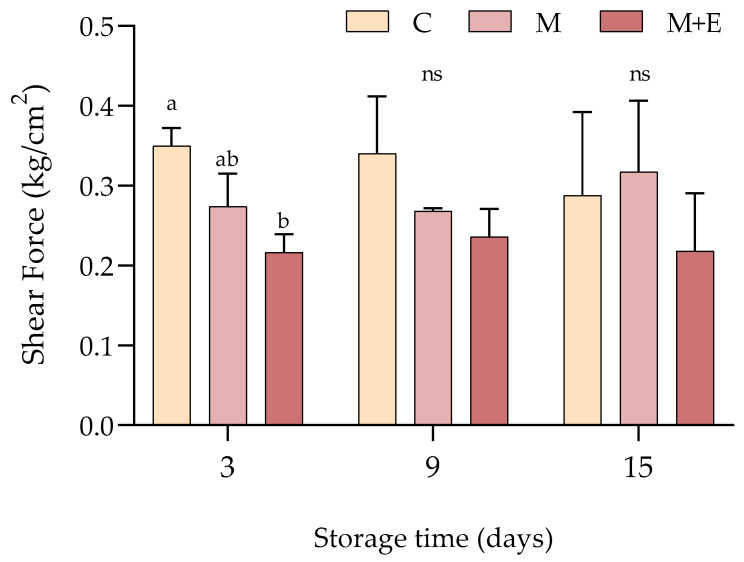
Average shear force values (kg/cm^2^) of non-marinated (C), marinated (M) and marinated with essential oils (M + E) pork loin slices at 3, 9 and 15 days of refrigerated storage. Data represent means ± SD. a, b = average values lacking a common letter significantly differ among the same sampling time. At the same storage time, ns indicates no significant differences among the samples.

Results concerning the shear force of cooked pork loin samples after 3, 9 and 15 days of refrigerated storage are displayed in Figure 5. After 3 days of refrigerated storage, non-marinated samples showed significantly higher shear forces than the marinated ones (M and M + E) (*p* < 0.05), with M + E group exhibiting the lowest values. Albeit no statistical difference has been detected at 9 and 15 days likely due to the high variability of data, M + E samples showed the lowest shear force values, thus suggesting that the effect of essential oils on improving meat tenderness is considerable in particular in the first days of storage.

#### 3.1.4. Sensory Analysis

**Figure 6:**
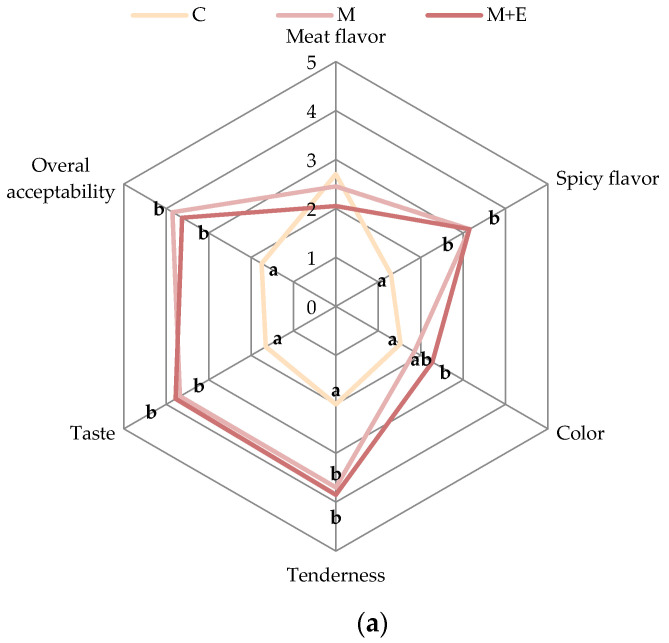

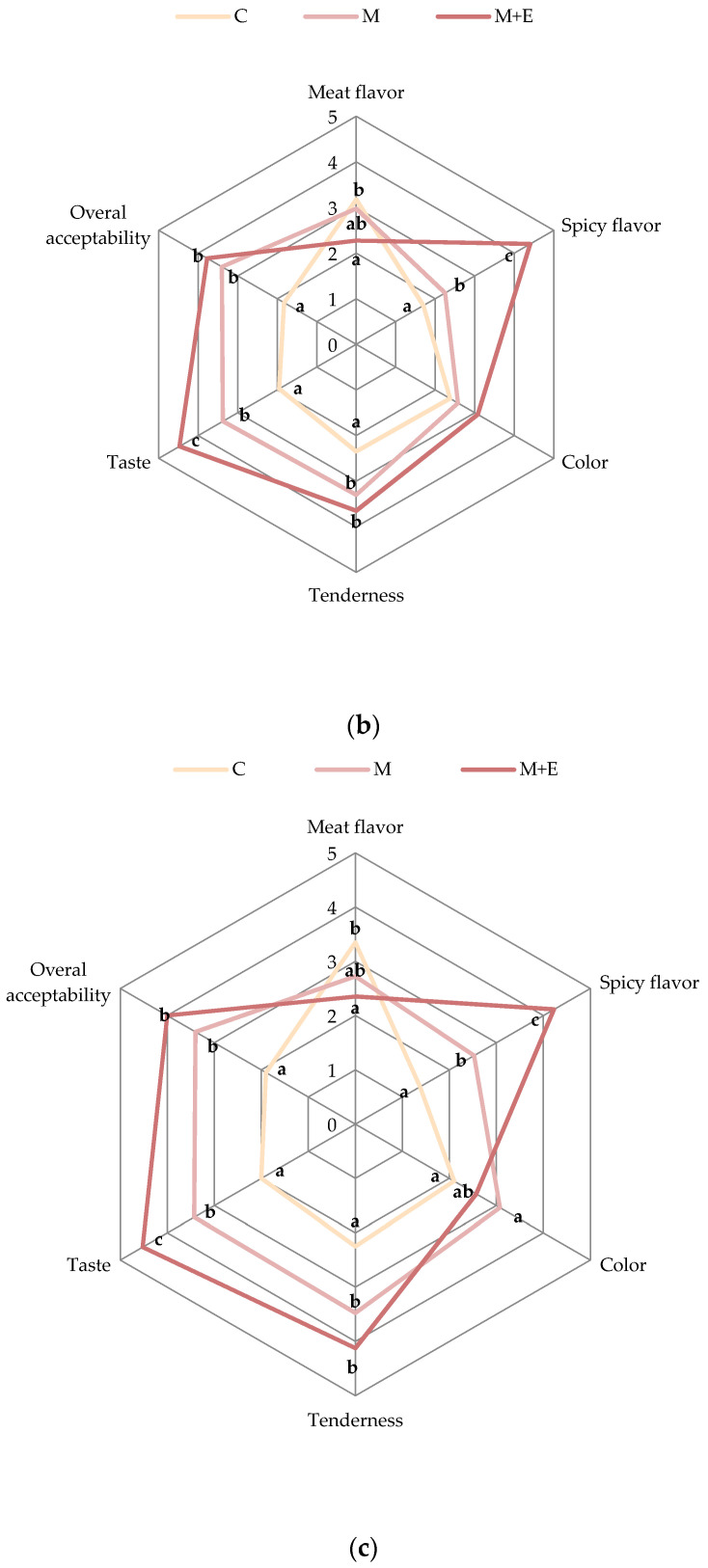
Sensory data of pork loin slices after 3 days (**a**), 9 days (**b**) and 15 days (**c**) of storage in relation to the sample (Control (C), Marinated (M), marinated with essential oils (M + E)). Data represent means ± SD. a, b, c = average values of each sensorial parameter lacking a common letter significantly differ among the same sensory parameter.

Panel tests were performed on pork loin samples after 3, 9 and 15 days of storage with the aim of determining the acceptability of the product by the consumers. The results of the panel tests are shown in Figure 6a–c.

The results showed that, regardless the sampling time, the marinated meat, and especially that with essential oils (M + E), exhibited better scores compared to the non-marinated one, with the only exception of meat flavor intensity parameter. In addition, the marinated samples showed a greater intensity of flavor and taste, positively perceived by the panelists. In particular, marinated meat slices showed higher tenderness, color, flavor and taste intensities for the whole storage period, resulting in an overall improved acceptability compared to the controls. Considering the effect of essential oils, no differences between M and M + E samples were observed after 3 days of storage. However, starting from the second panel test (day 9), M + E samples showed higher scores for spicy flavor and taste intensity compared to M samples. The differences among M and M + E samples intensified at the end of storage (day 15), when the M + E group showed the highest scores for overall acceptability, thus being the preferred sample for over 60% of panelists.

#### 3.1.5. Microbiological Analysis

The microbiological analyses were aimed to detect various microbiological groups frequently associated with the spoilage of processed meat products. In particular, during the refrigerated storage of the samples, the cell loads of total aerobic mesophilic and psychotropic bacteria, lactic acid bacteria, yeasts, *Pseudomonas* spp., total coliforms and *Brochotrix thermosphacta* were detected.

**Figure 7:**
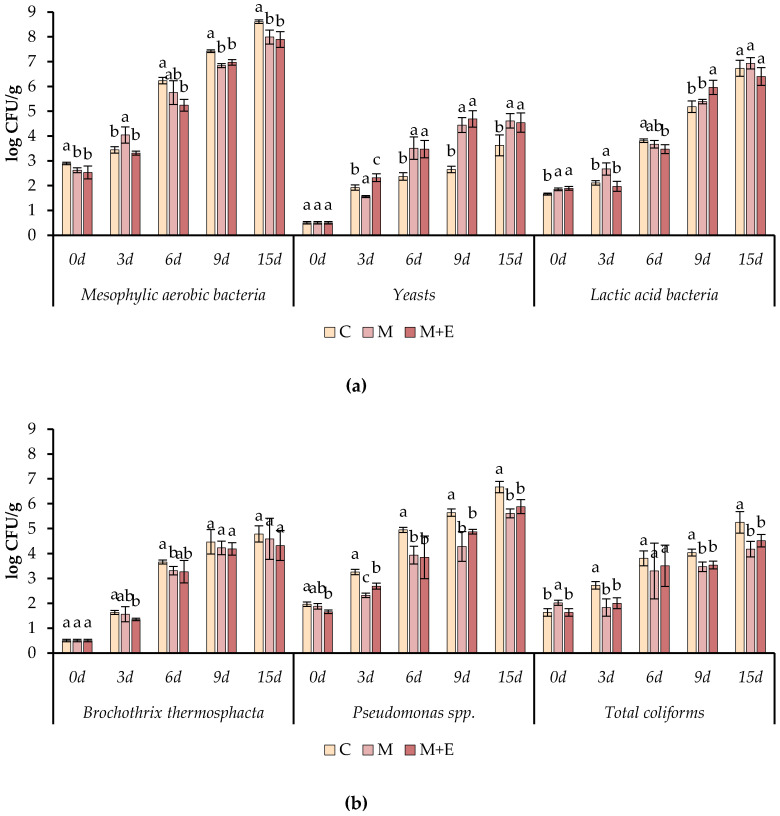
Cell load (log CFU/g ± SD), during the refrigerated storage, of total aerobic mesophilic bacteria, yeast and lactic acid bacteria (**a**) *Brochothrix thermosphacta*, *Pseudomonas* spp. and total coliforms (**b**) in different pork loin slices: Control (C), Marinated (M), marinated with essential oils (M + O). Data represent means ± SD. a-b-c = for each microorganism, at the same time of storage, average values lacking a common letter significantly differ among the same sampling time (*p* < 0.05).

In Figure 7, the cell loads of mesophilic aerobic bacteria, lactic acid bacteria, yeasts, *Pseudomonas* spp., total coliforms and *Brochotrix thermosphacta* are reported.

The data obtained indicated a satisfactory microbiological quality of the raw meat. In fact, the initial cell load of the main spoiling microorganisms was below 3.0 log CFU/g, independently on the use of marinade solution or the addition of essential oils. As expected, the mesophilic bacteria represented the main microbial spoiling group. In fact, a fast increase of the cell load of this group was observed in all the samples starting from the sixth day of refrigerated storage. However, from day 6 of storage, samples M and M + E showed significant lower cell loads compared to C, while no differences were observed between M and M + E samples. The C samples were the only ones found to exceed 8.0 log CFU/g after 15 d of storage. The same trend was observed for psychotropic aerobic bacteria.

A similar tendency was observed for *Pseudomonas* spp. Starting from day 3 of storage C samples showed significant higher cell loads compared to M and M + E samples. No significant differences regarding the cell load of *Pseudomonas* spp. were observed between M and M + E samples, with the only exception of day 3. At the end of the storage *Pseudomonas* spp. resulted 6.67, 5.61 and 5.88 log CFU/g respectively in C, M and M + E samples. Total coliforms resulted significantly lower in M and M + E samples compared to C ones, excepted at day 3 of storage. The greatest differences were observed at day 15 when coliforms were 5.25, 4.18 and 4.22 log CFU/g respectively in samples C, M and M + E. In general, the highest inhibition due to marination and the addition of essential oils was observed against the Gram-negative bacteria *Pseudomonas* spp. and total coliforms. Otherwise, minor differences were observed considering *B. thermosphacta* since no significant differences were observed starting from day 9 of storage. However, depending on the sample, this microorganism reached a cell load ranging between 4.4 and 4.8 log CFU/g.

A different trend was observed for yeasts and lactic acid bacteria. In fact, starting from day 6 of storage, yeasts resulted significantly higher in samples M and M + E compared to the control. However, yeasts never exceed 5.0 log CFU/g for the whole period of storage. In case of lactic acid bacteria, no significant differences were detected at the end of the storage among the samples.

### 3.2. Challenge Test

**Figure 8:**
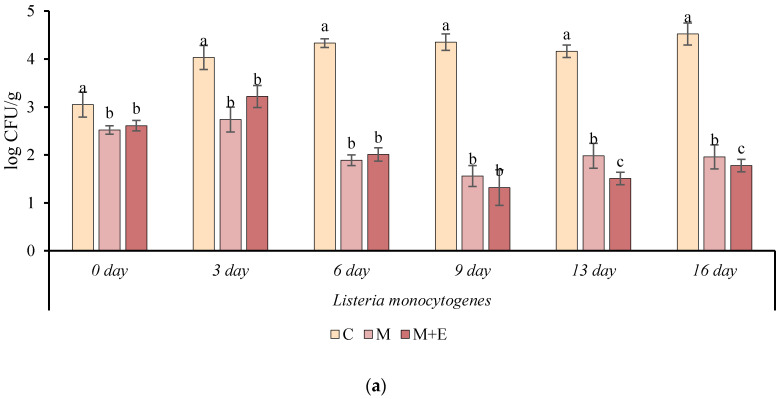

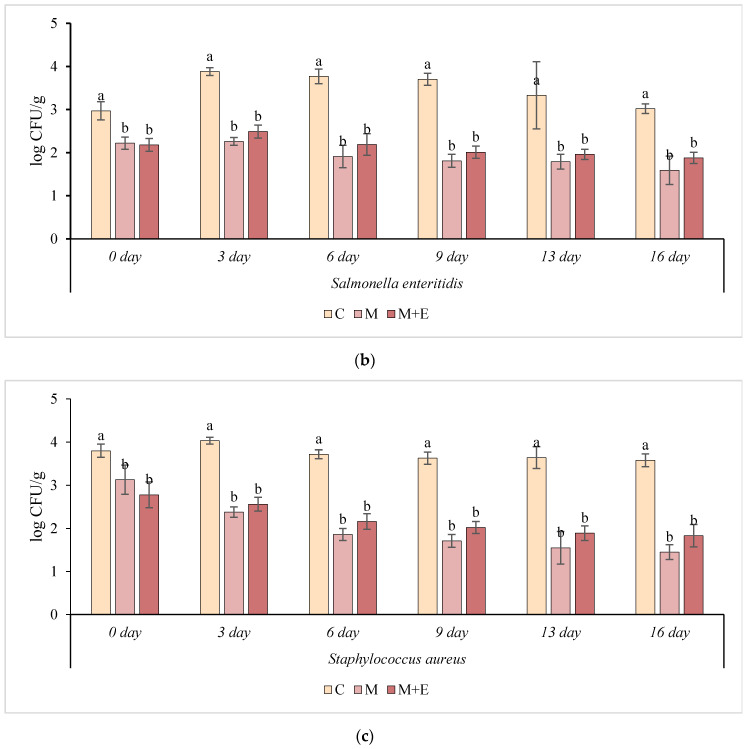
Cell load (log CFU/g ± SD), during refrigerated storage, of *Listeria monocytogenes* (**a**), *Salmonella enteritidis* (**b**), and *Staphylococcus aureus* (**c**). in different pork loin samples: Control (C), Marinated (M), marinated with essential oils (M + E). Data represent means ± SD. a, b, c = for each microorganism, at the same time of storage, average values lacking a common letter significantly differ among the same sampling time (*p* < 0.05).

In order to evaluate the effects of the marinade solution with or without essential oils on the safety of vacuum packed pork loin slices, a challenge test inoculating *Listeria monocytogenes* Scott A, *Salmonella enteritidis* E5 and *Staphylococcus aures* SR31 was performed. Figure 8a–c shows the cell loads of the pathogen microorganisms during the refrigerated storage.

It is noteworthy to mention that marination allowed a significant (*p* < 0.05) reduction of the initial microbial cell load of all the pathogens, regardless of the presence or absence of essential oils. The highest initial cell load reduction, compared to control samples, was observed for *S. aureus*, and ranged between 0.7 and 1.0 log CFU/g, followed by *S. enteritidis* (0.7–0.8 log CFU/g) and *L. monocytogenes* (0.5–0.6 log CFU/g). In all cases, the differences in the pathogen levels between marinated and not marinated samples increased during the storage period. At the end of the storage, M and M + E samples showed cell loads lower than 2.0 logarithmic cycles for *L. monocytogenes* and *S. aureus* and lower than 1.5 logarithmic cycles in the case of *S. enteritidis*. On the contrary, an increase of the level of all the pathogens in C samples, greater in the case of *L. monocytogenes*, was observed during storage. Contrarily, a decrease of the pathogen loads in the marinated products was observed during the storage but without allowing their complete inactivation. Considering the effect of the addition of essential oils, no significant differences were found between the samples M and M + E for *S. enteritis* and *S. aureus* while in the case of *L. monocytogenes* the samples M + E showed a significantly lower cell load with respect to samples M starting from day 13 of storage. The greatest antimicrobial effect from marinating was observed against *S. aureus*. In fact, a reduction of more than 3.5 log CFU/g at the end of storage compared to the initial load of C samples was observed.

## 4. Discussion

The marinade solution prepared with extra virgin olive oil, beer, concentrated lemon juice and a mixture of essential oils used within this study was selected based on the findings of preliminary trials. Considering that offering a marinated product including typical ingredients and flavors belonging to the Mediterranean diet may represent an added value to product itself, all the marinade ingredients and essential oils chosen in this work derive from plants commonly used in the traditional recipes of this area. The selected marinade solution was then tested with the aim of exploring its effect on the shelf-life, safety and quality traits of pork loin slices during refrigerated storage.

Aside from the inclusion of essential oils, the addition of the marinade solution significantly reduced the pH of vacuum-packed pork loin. These outcomes might be ascribed to the addition of an acid marinade solution in which the inclusion of beer (pH = 3.96) and concentrated lemon juice (pH = 2.26) results in a remarkable reduction in pH. This might be desirable for several reasons. First, meat pH exerts a direct effect on its water holding capacity (WHC), since it is generally held that the ability of meat to retain water progressively improves above and below pH values corresponding to the isoelectric point of meat proteins (i.e., 5.5 in the case of pork meat) [26]. Furthermore, processed meat products with a low pH are less likely to develop pathogen microbial growth and off-odors, thus having an improved safety and shelf-life [27,28]. Lastly, reduced pH values might also be advantageous to facilitate the action of collagenases and other proteolytic enzymes responsible for meat tenderization during the refrigerated storage [29].

The addition of the marinade solution, regardless of the use of essential oils, also exerted a significant effect on the yellowness (b*) of meat samples, while lightness (L*) and redness (a*) were not affected. The higher b* values detected for marinated samples might be likely due to the presence of coloring compounds in the solution itself (i.e., extra virgin olive oil, beer and concentrated lemon juice) which might have increased the yellowness of samples. However, the increase in b* values did not negatively affect the sensory evaluation by panelists who associated to the marinated samples in general, and to those including essential oils in particular, a better color retention if compared to the control.

Beside all, the marinating process is a widely used procedure at industrial level implemented with the aim to improve not only the sensory and eating qualities of meat products but also their technological properties, with a special reference to WHC [30,31]. Accordingly, satisfactory marinade uptakes (of more than 7%) were observed for both marinated pork loin groups after 3 days of storage. Albeit little literature is available concerning the effects of essential oils to improve the technological properties of meat, the remarkable improvement in marinade uptakes might be ascribed to the acid pH of the marinade solution. Indeed, as lemon juice contains citric acid, this ingredient is often included within the marinade solution to improve meat WHC by lowering its pH [32]. These outcomes are in agreement with those reported by other authors that observed a marinade uptake ranging between 4.6 and 9.7% in acidic marinated *Longissimus dorsi* muscles [33]. However, it is noteworthy to remember that the marinade uptake is strongly related to the meat type, marination technique as well as the duration of the process [34].

The marination process allowed to remarkably reduce the cooking losses compared to control samples after 3 days of refrigerated storage. This trend is in agreement with what reported by Gao et al. [35] who assessed the effect of marination on the main quality aspects of vacuum-packed pork loin meat. However, after both 9 and 15 days of storage, marinated meat (either M or M + E) exhibited slightly higher cooking losses if compared to the control group. This trend might be likely due to the greater marinade uptake measured during the storage period, which might have resulted in a higher loss of fluids during cooking. Therefore, it is reasonable that raw meat, added with salt without the inclusion of marinade solution, presented reduced cooking losses after a week of refrigerated storage.

Several authors have reported an increase in tenderness of marinated poultry, pork and beef [11,32,35]. Accordingly, the addition of marinade solution with or without essential oils allowed to reduce the shear forces of pork loin meat of about 40% and 22.8%, respectively, just after 3 days of refrigerated storage. These outcomes suggest the effectiveness of an acidic marinade solution to improve the tenderness of meat samples, as previously reported by Miller [36]. Accordingly, several studies have reported that acidic substances in the marinating solution (including lemon juice) can play a crucial role in the tenderization of marinated meat, leading to meat fibers swelling and enhancing proteolysis [37,38].

The sensory analysis data, according to the available literature, suggested that the marinated samples, and in particular those in which essential oils were added to the marinade, were tender and characterized by better color, flavor and taste intensity compared to the control samples. On the other hand, the positive effect of acidic marinade solutions on tenderness and other quality characteristics of different types of meat is widely reported in the literature [2,39]. The addition of essential oils strongly increased the overall acceptability of the samples, especially at the end of the storage, resulting in the preference of the consumers. Recently, many studies have reported an improvement of the sensory qualities and an extended shelf life of meat and meat products supplemented with different essential oils including, rosemary, thyme, oregano, basil, coriander, ginger, garlic, clove, juniper and fennel, used alone or in combination [40,41]. In addition, essential oils are widely reported as characterized by a strong antioxidant activity [42,43]. A wide literature reports a reduction of the lipid oxidation of meat and meat products added with essential oils during storage [40,44,45]. A better sensory quality and a longer shelf-life is normally associated to the reduction of lipid oxidation [45,46].

The predominant spoiling bacteria associated to refrigerated pork and beef, are *Pseudomonas* spp. during storage in aerobic conditions and lactic acid bacteria belonging to the genus *Lactobacillus* spp., *Leuconostoc* spp. and *Carnobacterium* spp. but also *Brochothrix thermosphacta*, *Enterobacteriaceae* and psychrophilic *Clostridium* spp. in case of anaerobic conditions [47,48]. Meat defects due to off-odors and off-flavors normally linked to a discoloration, gas production and acidification are generally associated to the growth of these microorganisms [49,50,51]. Our results indicate a satisfactory initial microbiological quality of the pork loin used in the present study. In fact, for all the main microbiological spoilage agents considered, the cell load was lower than 3.0 log CFU/g. During storage, an increase of the total viable mesophilic and psychotropic bacteria, *Pseudomonas* spp. lactic acid bacteria and *B. thermosphacta* was observed. The enumeration of total viable mesophilic and psychotropic microorganisms represents one of the most widely used and recognized criteria for evaluating the microbiological quality of meat [52]. Generally, the product is considered acceptable when the cell load of these microorganisms is lower than 7.0 log CFU/g [53] and this level is generally taken at industrial level as the upper threshold to determine the product expiry date. Our results showed that marinated samples overcome this limit only after 15 days of storage while control samples exceeded the limit after 9 days of refrigerated storage. The marination, regardless the addition of essential oils, showed the highest inhibition against the Gram-negative bacteria *Pseudomonas* spp. and total coliforms. Several literature data showed that species belonging to *Pseudomonas* and other psychotropic microorganisms are the predominant cause of alteration of fresh packaged meat [54]. Several *Pseudomonas* spp. are responsible for the formation of superficial patinas and off-flavor when their concentration reaches levels between 7–8 log CFU/g in chilled meat products [55].

Currently, foodborne outbreaks caused by foodborne pathogens transmitted from meat product still represent a significant public health challenge [56]. Considering the last 10–15 years the most important foodborne bacterial pathogens associated to meat belong to *Salmonella* spp., *Escherichia coli*, *Campylobacter jejuni* and *Staphylococcus aureus* [57,58,59]. Our results showed a clear inhibitory effect of the tested marinades on the growth kinetic of *Listeria monocytogenes*, *Salmonella enteritidis* and *Staphylococcus aureus* resulting in an increased safety of the product. In particular, the tested marinating solution proved an immediate inhibitory effect against all the pathogens. In addition, an increase of pathogens cell load during storage was observed in control samples, while the marinated products induced a more or less marked decrease of the pathogens load without allowing their complete deactivation. Regarding the addition of essential oils, a significant additional antimicrobial effect, compared to marinated samples, was observed only against *Listeria monocytogenes*. The antimicrobial activities of essential oils and their bioactive components are well known and reviewed in a wide literature even if strongly affected by microbial species, strains, and physico-chemical and process variables [17,18,21,60,61]. Although strain dependent and affected by application conditions, the greatest resistance of Gram-negative bacteria, due to the presence of the outer membrane acting as a barrier to hydrophobic molecules, to many essential oils is well known [62]. Among the Gram-positive bacteria, the very high resistance of *Staphylococcus aureus* to many stress factors and antimicrobials including essential oils and their components is well documented [62,63]. Also the action mechanisms of several essential oil components against many microorganisms, including the target microorganisms taken into consideration in the present research, have been clarified by molecular tools [64,65,66,67]. The limited antimicrobial effects of the essential oils in the present work is probably due to the masking effect of ethanol and its synergistic effects with low pH values and NaCl of marinade. In fact, as shown by Lanciotti et al. [68] studying the boundary between the growth and no growth of *Salmonella enteritidis*, *Bacillus cereus* and *Staphylococcus aureus* in the presence of different growth controlling factors through probabilistic models, the effects of ethanol on the limitation of growth of the considered species was significant also at concentration of about 1% and not merely additive with temperature and NaCl concentration. Also, the presence of organic acids and the pH reduction by marinade contribute to mask the effects of the essential oils on the target microorganisms considered [69].

Several authors have reported the antimicrobial effect of marinating solution components [10,12]. In particular the antimicrobial effect of some acidic marinade solutions containing alcoholic drinks is associated to the presence of ethanol but also to phenolic derivatives and organic acids, contributing the last to the reduction of the pH of the product [10,70,71]. In addition, the combination of organic acids, ethanol and sodium chloride can strongly inhibit several microorganisms including pathogens like *Salmonella*, *Listeria monocytogenes*, *Escherichia coli* and *Staphylococcus aureus* [72,73].

## 5. Conclusions

The results of the present study highlighted that the marination of pork loin slices using a solution (formulated with typical ingredients from Mediterranean area) with a mix of extra virgin olive oil, beer and lemon juice (in the presence/absence of essential oils) allows to obtain an overall improvement of the technological and sensory properties of meat. In particular, panel test results suggest a clear preference for marinated products with the addition of essential oils. Furthermore, the tested marinade solution exerted a remarkable meat pH reduction and significant antimicrobial activity both towards the common spoiling microflora normally present on the product and on pathogenic microorganisms deliberately inoculated, improving product safety and shelf-life. The use of marinade allowed the extension of the shelf-life of six days. In addition, offering a marinated product formulated with typical ingredients and flavors belonging to the Mediterranean diet may represent an added value to product itself. However, the addition of essential oils did not lead to a further increase of the antimicrobial activity exerted by the marinade solution. Though, the results obtained in this study suggest that an optimization of the concentration and type of essential oils used for the marination of pork loin could further increase its antimicrobial activity.

## Figures and Tables

**Table 1:** Marinade solutions tested in the preliminary trials.

Marinade Solution	Ingredients Ratio	% of Marinade Solution (*w/w*)
Water/lemon juice	1:1	10
Water/lemon juice	1:1	5
Olive oil/lemon juice	1:2	5
Olive oil/lemon juice	1:2	10
Olive oil/balsamic vinegar	1:1	5
Olive oil/balsamic vinegar	1:1	10
Olive oil/red wine	1:2	10
Olive oil/red wine	1:3	10
Olive oil/white wine	1:2	10
Olive oil/white wine	1:3	10
Olive oil/beer	1:2	10
Olive oil/beer	1:3	10
Olive oil/beer/lemon juice	1:2:1	10
Olive oil/mustard/lemon juice	1:1:1	10
Olive oil/mustard/lemon juice	1:1:1	5
